# Revisiting Transfer Functions: Learning About a Lagged Exposure-Outcome Association in Time-Series Data

**DOI:** 10.3389/ijph.2022.1604841

**Published:** 2022-07-11

**Authors:** Hiroshi Mamiya, Alexandra M. Schmidt, Erica E. M. Moodie, David L. Buckeridge

**Affiliations:** Department of Epidemiology, Biostatistics and Occupational Health, School of Population and Global Health, McGill University, Montreal, QC, Canada

**Keywords:** time-series analysis, lagged association, environmental exposure, transfer function, food marketing, sugar-sweetened food, dynamic linear model, Bayesian analysis

## Introduction

Environmental exposures often show a time-lagged association with outcomes [1–3]. Distributed lag models have been used to capture such lag patterns by incorporating time-lagged values of exposures, with the corresponding of the lag structure approximated by polynomials or splines [1, 4]. These models require the correct input of cut-off time, or pre-specified window (hereafter termed lag length), after which the association diminishes to a constant level, typically zero [5, 6]. However, lag length is often unknown [5–7]. To fit distributed lag models without specifying lag length, we revisit transfer functions (TFs), a method to specify time-lagged associations commonly used in econometrics and introduced to epidemiology in 1991 [8–10]. We provide a case study to capture the time-lagged association between weekly purchasing outcome of sugar-sweetened drinkable yogurt and weekly-varying display promotion of these beverages, which is an obesogenic food environmental exposure in supermarkets.

## Methods

TFs capture a time-lagged exposure-outcome association using a structural variable, denoted *E*_*t*_, which summarizes the current association (at time *t*) and cumulative association (up to time *t*) between the outcome variable *Y*_*t*_ and time-lagged exposure variable *X*_*t*−1_ + *X*_*t*−2_ + *X*_*t*−3_+... [8, 11] (Supplementary Appendix S1). We illustrate a simple form of TF to capture a commonly observed shape of lag pattern, a monotonically decreasing association of outcome and lagged exposure, often called the Koyck decay [12]. Using the decay coefficient of lagged association λ up to lag *h*, the decreasing associations are represented as

$$E_{t}=\beta X_{t}+\lambda^{1}\beta X_{t-1}+\lambda^{2}\beta X_{t-2}+\cdots +\lambda^{h}\beta X_{t-h}\text{,}$$

which recursively reduces to

$$E_{t}=\beta X_{t}+\lambda E_{t-1}\text{,}$$

$$E_{0}=0$$

The coefficient *β* captures the immediate association at time *t*, and the value of decay coefficient λ closer to 1 implies a more persistent association over time (i.e., slower decay), while a value closer to zero indicates a shorter lag [12, 13]. Constraining λ to be 0 < λ < 1 ensures the association monotonically decaying towards zero when the value of *β* is positive (Supplementary Figure S1A), and previous studies also imposed the decay towards zero [14, 15]. The variable *E*_*t*_ is added to a time-series regression for the outcome *Y*_*t*_ to estimate *β* and *λ* as *Y*_*t*_ = *E*_*t*_ + *Z*_*t*_ *γ* + *ε*_*t*_, where *Z*_*t*_ represents a set of covariates and intercept with coefficients γ, and *ε*_*t*_ represents the error term [10, 13].

**FIGURE 1:**
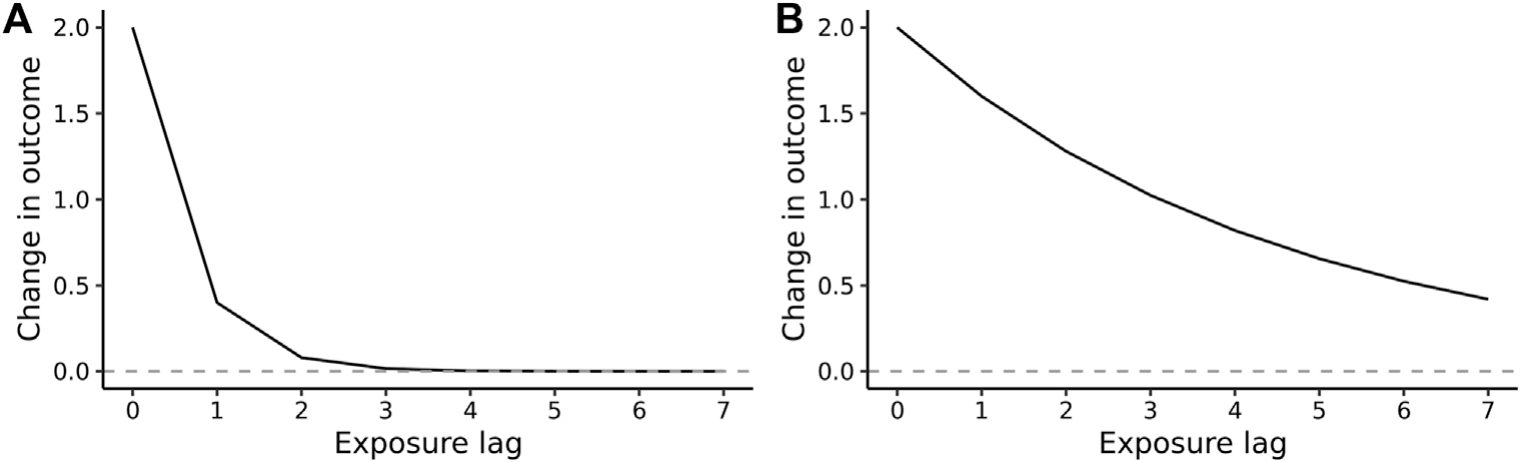
Hypothetical impulse response function of the Koyck lag transfer function, with the rate and extent of decay being controlled by the value of the lag parameter λ: **(A)** a weak decay returning to the baseline with a short lag (λ = 0.2): **(B)** a more persistent lag, i.e., slower decay (λ = 0.8). The value of the immediate effect, *β*, at the time of exposure (x = 0) is 2.0 in both plots (Hypothetical function, 2022).

A visual interpretation of a lagged association combining these coefficients is provided by an impulse response function (IRF), representing the change of the outcome *Y*_*t*+0_ + *Y*_*t*+1_ + *Y*_*t*+2_ + … + *Y*_*t*+*h*_ to an impulse (one-unit increase of x at time *t* only), while holding other variables constant [16]. The IRF of the Koyck decay is *β* + *βλ* ^1^ + *βλ* ^2^ + … + *βλ* ^*h*^, visualized in Figure 1.

**FIGURE 2:**
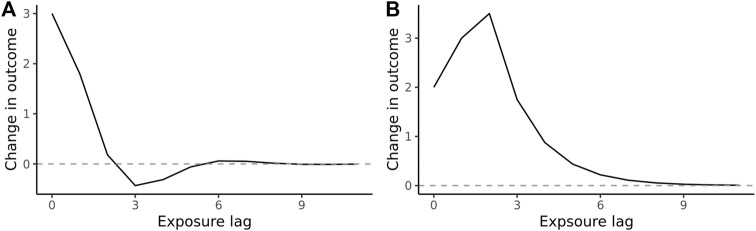
Hypothetical impulse response function of **(A)** short-term negative association (a “dip” below zero) following the decay of positive association and **(B)** delayed peak of positive association (Hypothetical function, 2022).

The general specification of the TF capturing various shapes of lag structure is

$$E_{t}=\beta_{0}X_{t-0}+\beta_{1}X_{t-1}\ldots \beta_{p}X_{t-p}+\lambda_{0}E_{t-0}+\lambda_{1}E_{t-1}+\ldots +\lambda_{q}E_{t-q}$$ (1)

where the Koyck decay is captured by *p* = 0, *q* = 1 in Eq. 1 above. More complex shapes are specified by higher values of *p* and *q* (Figure 2; Supplementary Appendix S2), allowing generalization to classical lag models, such as the Almon polynomial [10, 17].

Unlike commonly used distributed lag models, TF models obviates pre-specification of a lag length *h*, but require prior biological and epidemiological knowledge to help select plausible shapes of the lag (values of *p* and *q*). Deciding among candidate shapes is facilitated by model selection using fit metrics such as an information criterion [11].

## Case Study

The exposure is the weekly within-store display promotion of sugar-sweetened food items that potentially exhibits time-lagged association with the number of these items sold (outcome). Display promotion is the temporary placement of items in prominent locations to increase sales of (typically) ultra-processed food [18]. Our food of interest is sugar sweetened (not plain) drinkable yogurt, a hidden and important source of dietary sugar among children [19, 20]. A time series of weekly proportion of display-promoted sugar-sweetened drinkable yogurt items (continuous exposure) and weekly sum of the sales quantity of these items (continuous outcome) are recorded from a large supermarket in Montreal, Canada over *T* = 311 weeks (6 years). Supplementary Appendix S3 and Supplementary Figures S2, S3 elaborate the definition of the exposure and outcome.

The time-series regression used in this study is a dynamic linear model [21, 22]. We added the structural variable, *E*_*t*_, covariates, a seasonal term, and an intercept. We selected the Koyck lag TF (*p* = 0, *q* = 1) for *E*_*t*_, since the promotion exposure is likely to have a monotonically decaying association with purchasing [6]. The model was fit under the Bayesian framework as described in Supplementary Appendix S4.

**FIGURE 3:**
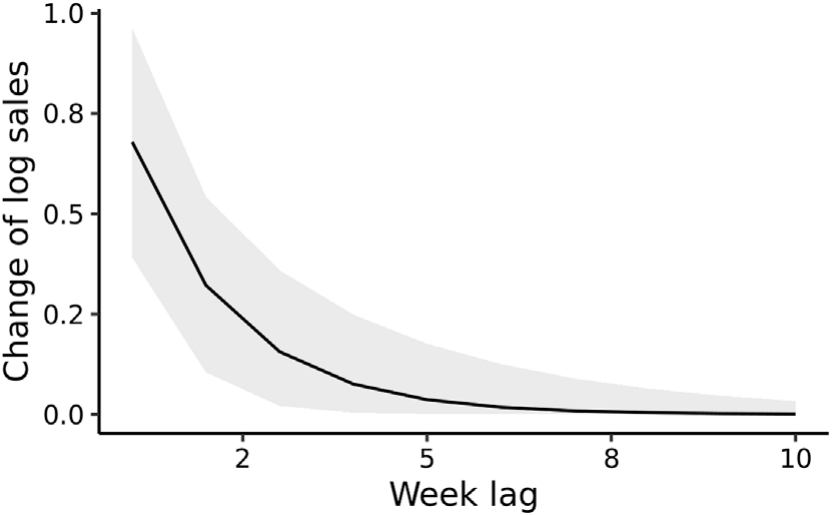
The estimated impulse response function of display promotion on the (natural log) sales of sugar-sweetened drinkable yogurt, based on the lag parameters *β* and λ learned from the time-series of sales data from a single store (Montreal, Canada, 2008–2013). The grey band indicates pointwise 95% posterior credible interval. The immediate association is displayed at lag 0 and is 0.68 (95% Posterior Credible Interval: 0.39–0.96), indicating that the immediate impact of display promotion is a doubling of sales, since exp(0.68) = 1.97.

The estimated immediate effect of the TF *β* was 0.68 (95% Posterior Credible Interval [CI]: 0.39–0.96), implying two-fold increase in sales at week *t*, if all yogurt items were display- promoted in the same week. The point estimate of the decay coefficient λ was moderately strong: 0.47 (95% CI 0.20–0.72), as shown by the distinct lag in the estimated IRF (Figure 3). Residual diagnostics indicate the absence of temporally autocorrelated residuals (Supplementary Figure S4).

## Discussion

Time-lagged exposure-outcome associations are of critical interest in time-series analysis. We described TF modeling to estimate lagged associations when lag length is unknown *a priori*. Previous applications of TFs include environmental time-series analysis to capture decaying associations between arbovirus incidence and temperature [23] and interrupted time-series analysis to capture the persistent effect of interventions [11, 24]. TF modeling requires pre-specification of the shape of a lag structure from investigators’ prior knowledge followed by their selection based on model fit. When such knowledge is lacking, existing distributed lag models such as those using splines allow data-driven estimation of the shape of lag. They require the specification of lag length by model selection applied to plausible lag lengths [25], by setting a long enough length to cover the unobserved true lag window with a potential sacrifice of precision [4], or alternatively estimating the lag length from data [26, 27]. Limitations of TFs include challenges in selecting the most appropriate shape of lag, when competing shapes show similar model fit. Finally, a comprehensive evaluation of TFs to capture lagged associations from simulated environmental health data is warranted, including their capacities to capture non-linear exposure-outcome associations by making β time-varying (dynamic) or imposing non-linear structure to *E*_*t*_ [17, 28].
