# Supplementary material for: Revisiting Transfer Functions: Learning About a Lagged Exposure-Outcome Association in Time-Series Data
Source: Int J Public Health. 2022 Jul 11;67:1604841. doi: 10.3389/ijph.2022.1604841 (PMC9336681; doi:10.3389/ijph.2022.1604841)
Supplement: Supplementary file 1 [file DataSheet1.docx]

**Supplementary File**

Revisiting transfer functions: Learning about a lagged exposure-outcome association in time-series data using transfer functions.

Contents

[Supplementary eAppendix 1: Additional explanation of Figure 1a and b in the main document to interpret the impulse response and transfer function capturing a monotonically decaying lagged exposure-outcome association. 2](#_Toc103333267)

[Supplementary eFigure 1. Impulse response function of transfer function capturing a) the Koyck decay function, b) increasing value of association, and c) oscillating function, all plateauing towards zero. 3](#_Toc103333268)

[Supplementary eAppendix 2: Additional description of Figure 2a and b in the main document showing the impulse response function of higher order transfer functions to capture more complicated shapes of time-lagged exposure-outcome associations. 4](#_Toc103333269)

[Supplementary eAppendix 3: Definition of exposure and outcome 5](#_Toc103333270)

[Supplementary eFigure 2. 6](#_Toc103333271)

[Supplementary eFigure 3. 6](#_Toc103333272)

[Supplementary eAppendix 4: Specification of time-series regression and software codes 7](#_Toc103333273)

[Supplementary eTable 1. Description of time-series model containing transfer function. 8](#_Toc103333274)

[Supplementary eFigure 4. 17](#_Toc103333275)

[References for Supplementary Materials 18](#_Toc103333276)

# Supplementary eAppendix 1: Additional explanation of Figure 1a and b in the main document to interpret the impulse response and transfer function capturing a monotonically decaying lagged exposure-outcome association.

Briefly, a transfer function is a function that distributes the effect of a unit change in an exposure variable on the values of an outcome variable over multiple future time points. A time-series model containing transfer function is called transfer function model and can be generalized to several distributed lag models (1). Widely used in econometrics, the transfer function has been integrated into classic time-series models such as Autoregressive Integrated Moving Average models (ARIMA) (2) and dynamic models used in our case study (1,3). The ARIMA-transfer function model in intervention analysis was introduced into epidemiology in 1991 (4) and recently received renewed attention due to its ability to capture the complex effect of population interventions on outcomes over time in interrupted time-series studies (5). Depending on the pre-specified values determining the shapes of lag structures (*p* and *q* parameters in the main text) and the estimated values of decay ($\lambda$) and effect ($\beta$) coefficients from data, transfer functions can capture distributed lags, such as the monotonically decaying infinite lag model (Koyck) and the Almon polynomial model (1).

Interpretation of a time-lagged association captured by a transfer function model is typically visualized by the corresponding Impulse Response Function (IRF). Figure 1a and 1b in the main text show the IRF of the example transfer function capturing the Koyck decay, with the varying extent of decay controlled by the lag parameter $\lambda$.

In these figures, the y-axis represents the lagged association between the exposure and outcome. The exposure in these plots represents a one-time impulse exposure; its value is increased by one unit at lag 0, then set to zero at subsequent lag points. Constraining the value of $\lambda$ to $0 <\lambda<1$ in the example specification of transfer function in the main document ensures that the lagged association decays towards zero, and the decay is monotonic when the value of $\beta$ is positive. Constraining the lagged association to reach zero has been proposed in previous studies, as the impact of many environmental stressors dissipates over time (6,7). We note that an analyst can estimate the value of $\lambda$ from data without such constraint, or impose the constraint if there is a prior belief for the diminishing association toward zero. Transfer functions can also be specified to decay towards non-zero values, thus capturing the potential shift of the baseline outcome to a new level due to population interventions that often lead to permanent (sustained) effect (2,5).

As described in the main text, the IRF of the transfer function ($p=0, q=1$) specified to capture monotonically decaying associations over exposure lag length $h$ is $\beta\lambda^{0}+ \beta\lambda^{1} + \beta\lambda^{2} +\ldots+\beta\lambda^{h}$, when $0 <\lambda<1$. In other words, fixing all other variables constant, values of the outcome $Y$ by *h* periods, denoted as $Y_{t+h},$ following the one-time exposure at time *t* is shown as

$$Y_{t+0}=\beta$$

$$Y_{t+1}=\beta\lambda$$

$$Y_{t+2}=\beta\lambda^{2}$$

$$\vdots$$

$Y_{t+h}=\beta\lambda^{h}$,

where $Y_{t+0}$ is the immediate association $\beta\lambda^{0}= \beta$ during the period of exposure at time *t*, or $x=0$in the Supplementary eFigure 1a . On the other hand, negative values of $\beta$ with the same constraint to $\lambda$ result in the IRF increasing and plateauing towards zero as seen in eFigure1b. As well, negative values of $\lambda$ in the range of $-1<\lambda<0$ lead to an oscillating function as seen in eFigure 1c, which is not a relevant lag structure in population exposures in public health.


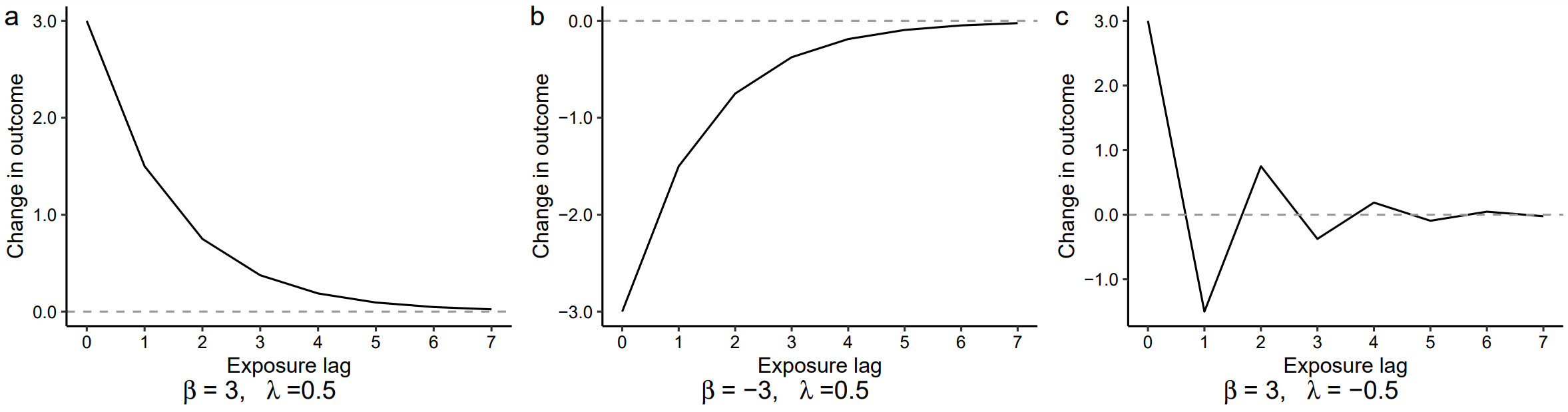


# Supplementary eFigure 1. Hypothetical impulse response function of transfer function capturing a) the Koyck decay function, b) increasing value of association, and c) oscillating function, all plateauing towards zero (Montreal, Canada, 2008-2013).

# Supplementary eAppendix 2: Additional description of Figure 2a and b in the main document showing the impulse response function of higher order transfer functions to capture more complicated shapes of time-lagged exposure-outcome associations.

The shape of the lagged association in Figure 2a is captured by a transfer function containing a second order autoregressive term. This form of transfer function, $p=0, q=2$, is the addition of another lagged structural variable $E_{t-2}$ with the corresponding lag coefficient $\lambda_{2}$ as follows

$E_{t}= \beta X_{t} +\lambda_{1}E_{t-1} + \lambda_{2}E_{t-2}$.

The corresponding Impulse Response Function (IRF) is of the form

$$Y_{t+0}=\beta$$

$$Y_{t+1}=\beta\lambda_{1}$$

$Y_{t+2}=Y_{t+1}\lambda_{1}$+ $Y_{t+0}\lambda_{2}$

$$\vdots$$

$Y_{t+h}=Y_{t+h-1}\lambda_{1}+ Y_{t+h-2}\lambda_{2}$,

where the coefficient of the immediate association is $\beta= 3.0$ and the lag coefficients are $\lambda_{1}=0.6$ and $\lambda_{1}=-0.3$ in Figure 2a. The short-term negative association is often termed mortality displacement in the time-series analysis of climate-associated mortality (e.g. heatwave) or called post-promotion dip in consumer marketing science (8,9).

In Figure 2b, the delayed peak after the impulse exposure period at $x=0$ is captured by a transfer function of form $p=2, q=1$. In this form, two variables with lag 1 and lag 2 from the same exposure ($X_{t-1}$, $X_{t-2})$ and the corresponding coefficients ($\beta_{2}, \beta_{3}$) are added as follows:

$E_{t}= \beta_{1}X_{t} +\beta_{2}X_{t-1}+\beta_{3}X_{t-2}+\lambda E_{t-1}$.

The corresponding IRF is a polynomial of the form:

$$y_{t+0}=\beta_{1}$$

$y_{t+1}=\beta_{1}\lambda^{1}$ + $\beta$

$$y_{t+2}=\beta_{1}\lambda^{2}+\beta_{2}\lambda^{1}+\beta_{3}$$

$$y_{t+3}=\lambda y_{t+2}$$

$$\vdots$$

$y_{t+h}={\lambda y}_{t+h-1}$ .

In Figure 2b, the coefficient of the immediate association is$\beta_{1} =2, \beta_{2}=2, \beta_{3} =1$ and the lag coefficients are $\lambda=0.5$.

# Supplementary eAppendix 3: Definition of exposure and outcome

The food category of interest is sugar-sweetened drinkable yogurt, which is classified as ultra-processed food (10) and considered to be the source of excess sugar intake, along with other sugar-sweetened beverages such as soda (11). We excluded plain yogurt items that contain intrinsic sugar only (i.e., fructose), as they are classified as minimally processed food and thus non-sugar-sweetened. Weekly beverage transaction and promotion data in this study were purchased from a global marketing company, Nielsen, that collects barcode-scanned electronic transaction records from chain retail food outlets (12).

The outcome is the weekly sum of the sales of sugar-sweetened drinkable yogurt items sold in a large supermarket in Montreal, Canada between January 1^st^ 2008 and December 31^st^ 2013 (n=311 weeks). There were 29 distinct sugar-sweetened yogurt items that were display promoted at least once during the study period. Sold quantities of individual items were summed at each week and natural log-transformed to approximately follow a normal distribution. The descriptive plot of sales is provided in Supplementary eFigure 2 below.

Exposure is the aggregated weekly display promotion status of sugar-sweetened drinkable yogurt items. Display promotion is the form of in-store marketing that temporarily places selected food items to prominent locations, such as the store entrance, at the end of aisle, or at the checkout aisle in order to increase awareness of promoted items (13). The weekly time-series of display promotion was defined as the proportion of displayed items in that week among sweetened drinkable yogurt items (thus ranging from 0 to 1), as shown in Supplementary eFigure 3 below.


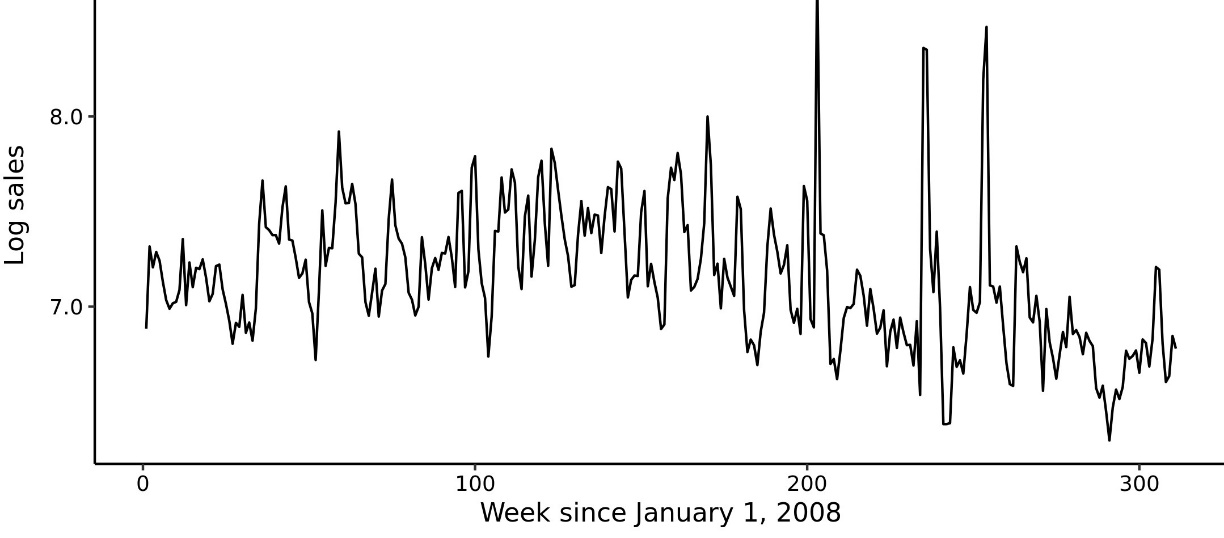


Supplementary eFigure 2. Natural log-transformed weekly sales of 29 yogurt items in a single supermarket, Montreal, Canada between January 2008 and December 2013 (Montreal, Canada, 2008-2013).


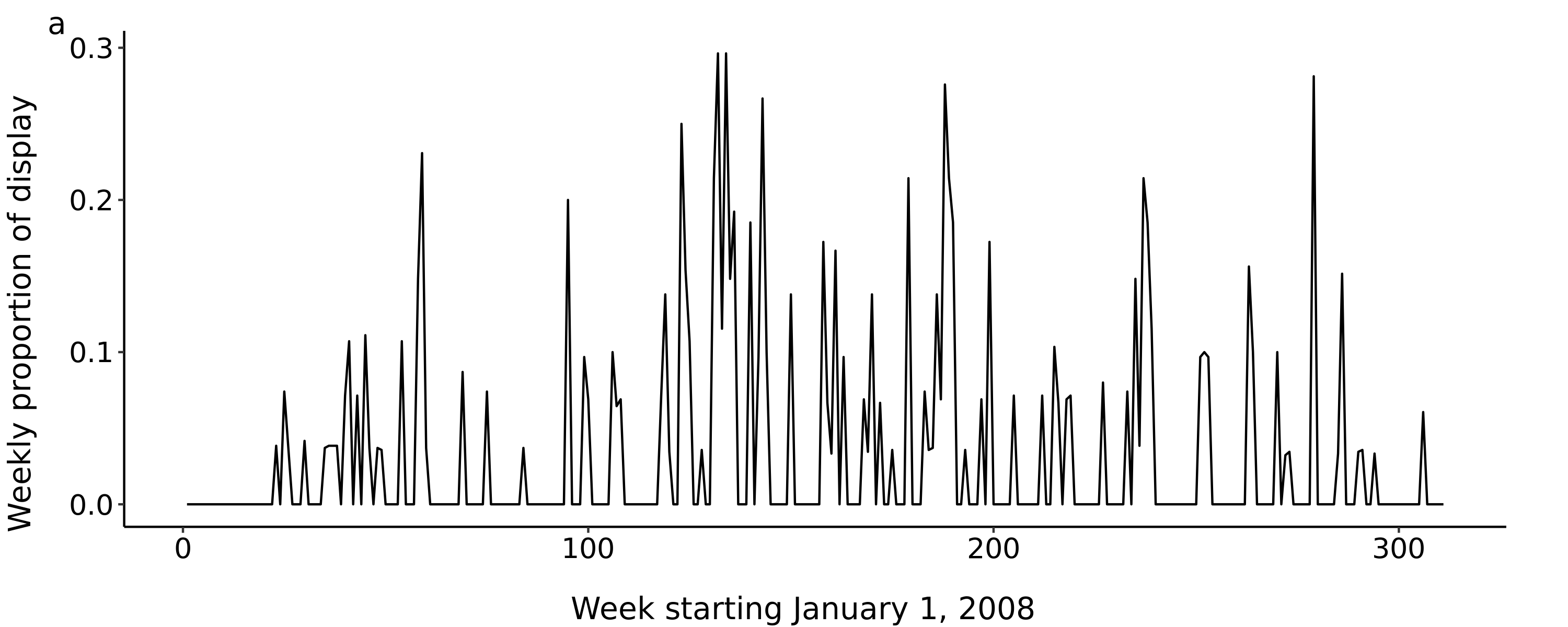


Supplementary eFigure 3. Weekly proportion of 29 yogurt items in a single supermarket, Montreal, Canada between January 2008 and December 2013 (Montreal, Canada, 2008-2013).

# Supplementary eAppendix 4: Specification of time-series regression and software codes

The structural variable capturing a transfer function, $E_{t}$, was added to a time-series model under the framework of dynamic linear model, a group of time-series regressions that allow regression coefficients to change smoothly (‘dynamic’) over time (3,14). The outcome in our case study, denoted $y_{t}$, is the natural log-transformed quantity of sales at week *t* and assumed to be normally distributed with mean $\mu_{t}$ and standard deviation $\sigma_{\epsilon}$ and modeled as:

$$y_{t}=\alpha_{t}+S_{t}+\delta C_{t}+E_{t} + \epsilon_{t},$$

where $\alpha_{t}$ is a time-varying (dynamic) intercept capturing trends and local fluctuation of outcome, $S_{t}$ is seasonal terms with a periodic cycle, and $C_{t}$ represents a vector of covariates with the corresponding time-fixed vector of coefficients $\delta$.

The local shift of the intercept at time *t*, $\alpha_{t}$ is determined by its previous value, $\alpha_{t-1}$, and random noise term $\epsilon_{alpha\_t}$; thus $\alpha_{t}=\alpha_{t-1}+\epsilon_{alpha\_t}$. The noise term, $\epsilon_{alpha\_t}$, follows a zero-mean independent normal distribution $\epsilon_{alph{a\_}_{t}}\sim Normal\left( 0,\sigma_{\alpha}^{2} \right)$ with the smoothness controlled by $\sigma_{\alpha}$.

The seasonal component $S_{t}$ is a harmonic representation of time with a sinusoidal wave. The periodicity is defined as $2\pi t\omega$, where $t=\{1,2, 3, \cdots,311\}$ as the week indicator, $\omega$ = 52.2 as the scalar representing the cycle (number of weeks in a year) and $\pi$ = $3.1415..$.. Regression coefficients, $\gamma_{Cos}$ and $\gamma_{Sine}$, capture the amplitude of the seasonal association as follows:

$S_{t}=\gamma_{Cos}Cos\left( 2\pi t\omega\right)+\gamma_{Sine}Sine\left( 2\pi t\omega\right)$.

Note that $\gamma_{cos}$ and $\gamma_{sine}$ are set to be time-fixed in this example (i.e., not dynamic) unlike the intercept.

As stated above, $C_{t}$ is the vector of weekly-varying covariates known to temporarily correlate with display promotion and sales. The covariates include weekly price displaying as defined in our previous study (blinded reference), consumer price index as the indicator of inflation, flyer promotion defined in the same manner as display promotion, and the binary indicator of week containing provincial and national statutory holiday. The corresponding set of time-fixed regression coefficients are $\delta$. Selection of the covariates were guided by Watanabe-Akaike Information Criterion (WAIC) that indicates a better model fit when its value is lower relative to the value of comparison models (15). We again note that the regression coefficients $\delta, \gamma_{cos}{and \gamma}_{sine}$ are time-fixed (non-dynamic regression coefficients) unlike the intercept. This represents a particular case of a dynamic linear model wherein some of the coefficients are fixed over time.

The parameters above were estimated following the Bayesian paradigm. Model specification is complete after assigning a prior distribution to the parameters of the model. The prior specification is described below. The resultant posterior distribution does not have a closed form. We resort to Markov chain Monte Carlo (MCMC) methods to obtain samples from the resultant posterior distribution. In particular, we used the rstan library in R software (16) which performs the MCMC using Hamiltonian Monte Carlo algorithms. We ran 3,000 iterations as a burn-in sample, and the following 30,000 iterations were used to simulate from the posterior distribution of the parameters. Convergence of the MCMC was inspected visually on the trace plots of the parameters from 3 independent chains and numerically based on the values of the effective number of sample size and Rhat for each model. Codes are available at the end of this appendix and in an online repository (https://github.com/hiroshimamiya/promotionLag).

Taken together, our time-series regression is structured as follows:

# Supplementary eTable 1. Description of time-series model containing transfer function (Montreal, Canada, 2008-2013).

| **Description** | **Model component** |
| --- | --- |
| Mean of outcome | $\mu_{t}$ $=\alpha_{t}+S_{t}+\delta C_{t}+E_{t}$ |
| Transfer function | $E_{t}= \beta X_{t}+\lambda E_{t-1}$  $E_{1} = \beta X_{1}$ |
| Time varying level | $\alpha_{t}=\alpha_{t-1}+\epsilon_{alpha\_t}$  $\epsilon_{alpha\_t} \sim Normal(0, \sigma_{\alpha}^{2})$ |
| Covariates | $\delta C_{t}$ |
| Seasonal effects | $S_{t}=\gamma_{Cos}Cos\left( 2\pi t\omega\right)+\gamma_{Sine}Sine\left( 2\pi t\omega\right)$ |
| Error term | $\epsilon_{t}\sim Normal\left( 0,\sigma_{\epsilon}^{2} \right)$ |

With the following priors:

$$\alpha_{1}\sim Normal(0,5^{2})$$

$$\sigma_{\alpha}\sim half-Normal(0, 1)$$

$$\sigma_{\epsilon} \sim half-Cauchy(0,5^{2})$$

$$\lambda\sim Uniform(0,1)$$

$$\gamma_{cos}{,\gamma}_{sine},\beta\sim Normal\left( 0,5^{2} \right)$$

$$\delta\sim Normal\left( 0,5^{2} \right)$$

The prior for the standard deviation of the error term was specified as a non-informative (i.e., diffuse) half-Cauchy distribution, that is,$\sigma_{\epsilon}\sim half-Cauchy(0, 5^{2})$ as previously suggested (17), with the constraint to take a positive value (i.e., constrained to the positive half of the distribution). To investigate whether posterior distribution is affected by the scale (diffuseness) of this distribution, we also ran models with a larger and smaller scale, $\sigma_{\epsilon} \sim half-Cauchy(0, {10}^{2})$ and $\sigma_{\epsilon} \sim half-Cauchy(0, 3^{2})$, respectively, which generated nearly identical posterior distributions. We also used a positive-constrained normal distribution to examine posterior sensitivity with the same values of the scaling parameter as above, which led to nearly identical results.

The prior probability of the time-fixed seasonal coefficients ($\gamma_{cos}{,\gamma}_{sine}$), each element of the coefficient set ($\delta$), and the coefficient of the immediate effect of the exposure ($\beta$) was specified as independent diffuse normal distributions as$Normal(0,5^{2})$. Again, we experimented with a larger value of the variance, $Normal(0,{10}^{2})$, and a smaller value, $Normal(0,3^{2})$, resulting in nearly identical posterior distributions. For the time-varying intercept, $\alpha_{t}$, the prior probability of the initial value at $t = 1$ was $\alpha_{1}\sim Normal(0,5^{2})$, which was subsequently allowed to evolve with a random noise whose standard deviation is $\sigma_{\alpha} \sim half-Normal(0,1)$, with posterior sensitivity analysis under alternative values of $\sigma_{\alpha} \sim half-Normal(0,0.1)$ and $\sigma_{\alpha} \sim half-Normal(0, 0.5)$. The small values of the standard deviation for the time-varying intercept allow a smooth transition of its parameters over time. The lag parameter was assigned a non-informative uniform distribution $\lambda\sim Uniform(0,1)$, which ensures the monotonic decay of the association towards zero as stated above.

We provide R codes to generate simple time-series data generated by the time-varying (dynamic) intercept, season effect, and the Koyck lag function to which the transfer function with the dynamic regression was applied to recover the lag function.

These materials are also found in an online repository: <https://github.com/hiroshimamiya/promotionLag>

------------------------------------------------------------------------

output: github_document

------------------------------------------------------------------------

# Script to generate sample data with time-lagged exposure-outcome association captured by transfer function implemented under dynamic regression framework

# Data are generated by R, and the model is ran by Stan software, via rstan library.

# Stan performs Bayesian inference using Markov Chain Monte Carlo

# See https://mc-stan.org/users/interfaces/rstan

# Clean environment

rm(list=ls())

# Load required libraries

require(rstan)

require(ggplot2)

# Provide multicore sampling to Stan, if a computer has multiple

options(mc.cores=parallel::detectCores())

# Fix random seed

set.seed(101)

# Function to generate outcome from lagged exposure-outcome association

# Function to generate matrix of exposure with lag, borrowed from : https://github.com/alastairrushworth/badlm/blob/master/R/lag_matrix.R

lag_matrix <- function (exposure, p, start.at.zero = T){

windows <- function(n) rev(exposure[n:(n + p - !start.at.zero)])

nums <- as.list(1:(length(exposure) - p))

matrix(unlist(lapply(nums, windows)), nrow = (length(exposure) - p), ncol = p + start.at.zero, byrow = TRUE)

}

### Example data ----------------------------------------------------------------

Tn <- 300 # number of time periods

# Lag decay coefficient

lambda <- 0.5

# Immediate effect

beta = 1

# Observation noise, standard deviation of error for Y

sigma_Y <- 1

# Intercept, e.g. log store-level sales of junk food, alpha_1 (initial value) is log(10)

# Standard deviation of shift in intercept

sigma_alphaLevel <- 0.2

alpha <- cumsum(rnorm(n = Tn, sd = sigma_alphaLevel)) + 10

# Generate season wave

weekIndex <- 1:Tn # this is time indicator, same as "t" variable in Supplemetary description of the model

freq <- 52.2

amplitude <- 2 # To be captured by the season coefficients

w <- 2*weekIndex*pi/freq

tempEffect <- amplitude*sin(w) + amplitude*cos(w)

# Alternatively, one can add temperature effect attached, weather in Montreal Canada between 2008 and 2013

#weather <- readRDS("./weather.rds")

#tempEffect <- as.numeric(weather$day_temp)*0.1

#tempEffect <- tempEffect[-(1:(length(tempEffect) - Tn))] # aligh number of time points

# Exposure with lag effect

H <- 0:20 # Horizon, max time window to define lag

lagFunction <- beta*lambda^(H) #exponential decay

#lagFunction <- (beta*exp(-(1-lambda)*H)) # more gradual decay

# Exposure, autoregressive order 1

x <- arima.sim(model = list(ar = 0.6), n = Tn+max(H), sd = 0.1)

x <- scale(x)[,1]

# Lagged effect at each time period

lag_mat <- lag_matrix(x, p = max(H))

# Generate outcome, combination of lagged effect of x, intercept, season and noise

yMean <- as.numeric(lag_mat %*% lagFunction) + alpha + tempEffect

y <- yMean + rnorm(Tn, mean = 0, sd = sigma_Y)

T <- Tn # Time variable,

x <- x[-H] # Trim lag horizon from the exposure

### plot

dev.off()

par(mfrow=c(2,2))

plot(lagFunction, type = "l", main = "Lag function", xlab = "Time lag", ylab = "Association")

plot(x, main = "Exposure", type = "l", xlab = "time")

plot(tempEffect, main = "Season wave (52 weeks frequency)", type = "l", xlab = "time")

plot(y, main = "Outcome", type = "l", xlab = "time")

par(mfrow=c(1,1))


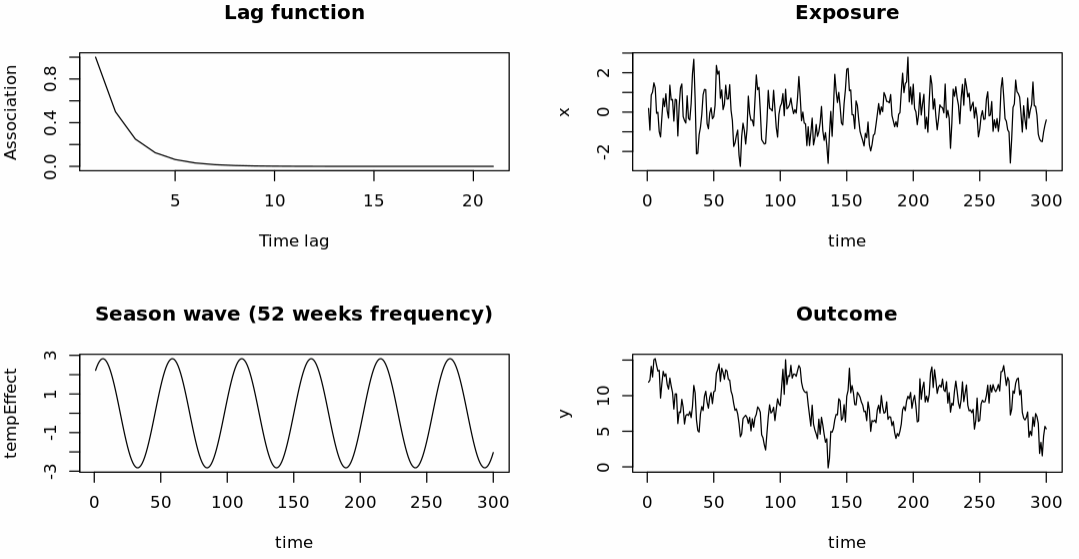


###Run-------------------------------------------------------------------------

# Fitting time-series model

fit3 <- stan(file="testModel_alphaLevel_season_koyck.stan", data=c("T","y","x", "weekIndex"),

iter=30000, chains=3, control = list(max_treedepth = 15))

# Quick mcmc diagnosis - Sign of convergence needs to be checked before interpretation of results

# See here: https://cran.r-project.org/web/packages/rstan/vignettes/rstan.html

traceplot(fit3, pars = c("sigma_Y", "sigma_alpha", "beta", "lambda", "alpha[2]", "alpha[200]", "alpha[300]", "gammaCos", "gammaSin")) +

ggtitle("Trace of MCMC")

pairs(fit3, pars = c("sigma_Y", "sigma_alpha", "sigma_alpha", "beta", "lambda", "alpha[2]", "alpha[200]", "alpha[300]")) +

ggtitle("Bivariate distribution of MCMC")

sum(summary(fit3)$summary[,"Rhat"] > 1.01)

# further checks

source("https://raw.githubusercontent.com/betanalpha/knitr_case_studies/master/stan_intro/stan_utility.R")

check_all_diagnostics(fit3)

# Autocorrelation function to see residual autocorrelation is not severe

fittedPosterior <- rstan::extract(fit3, pars = "yHat")

resids <- apply(fittedPosterior$yHat, 2, mean) - y

acf(resids, main = "Autocorrelation function of residuals")

hist(resids, main = "Histogram of residuals")


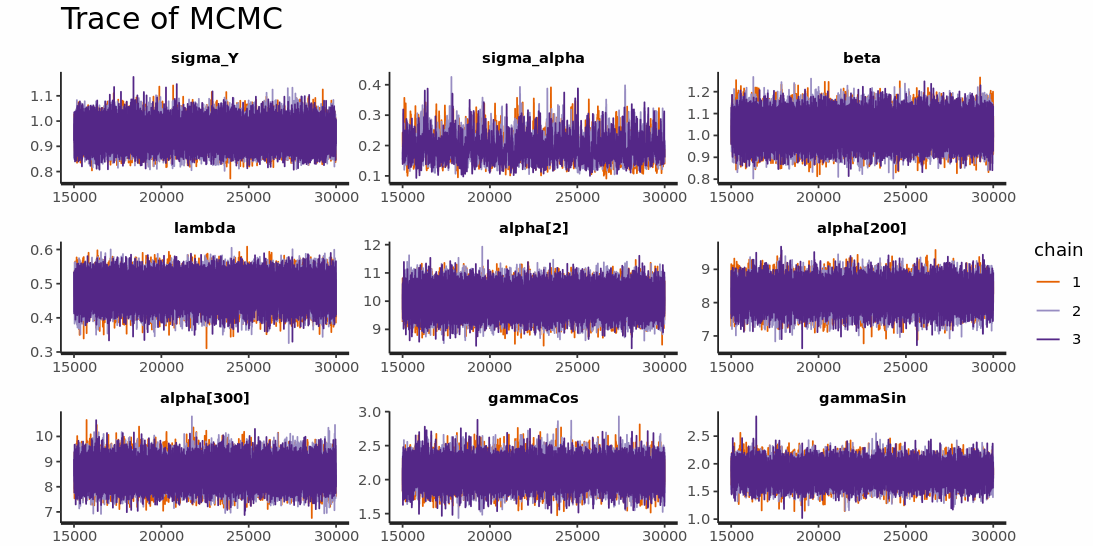


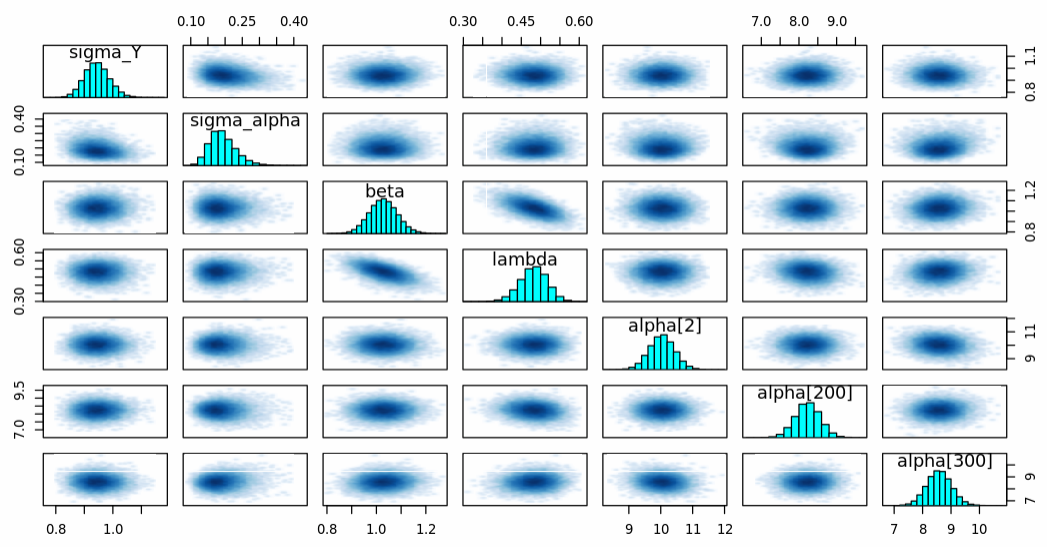


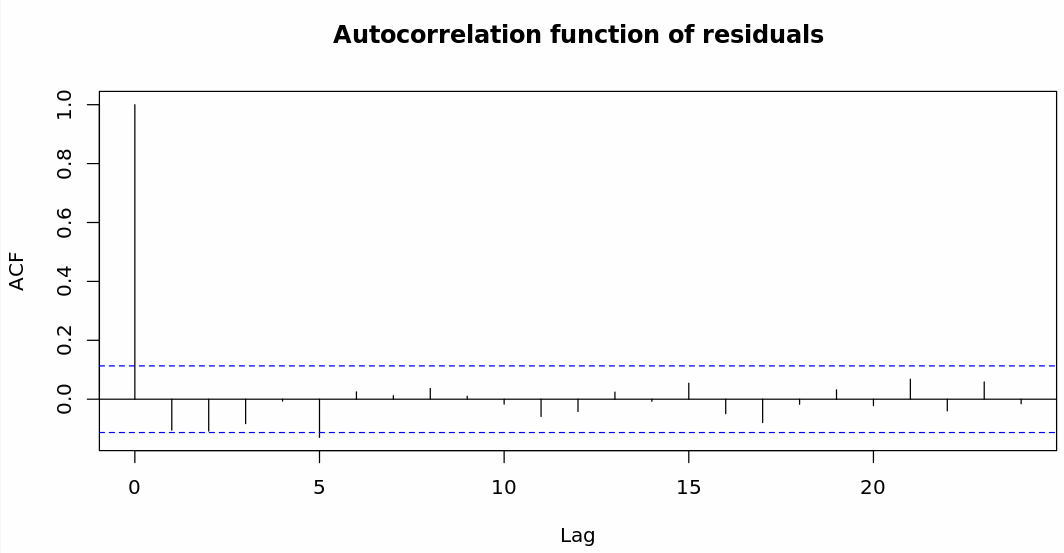

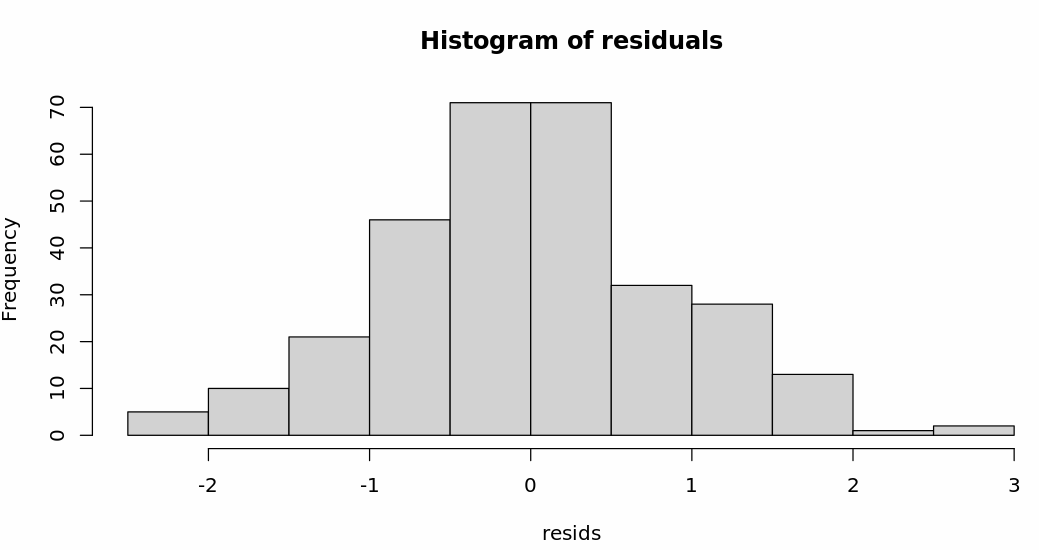


### Results --------------------------------------------------------------------

# Check estimated model paramters

plot(fit3,

ci_level = 0.95,

pars = c("beta", "lambda", "sigma_alpha", "sigma_Y", "gammaCos", "gammaSin")) +

ggtitle("Summary of recovered parameters (Posterior mean and 95% Interval)",

subtitle =

expression("These parameters correspond to " ~ beta ~ lambda ~ sigma[alpha] ~ sigma[epsilon] ~ gamma[cos] ~ gamma[sin] ~ "in Supplementary appendix"))


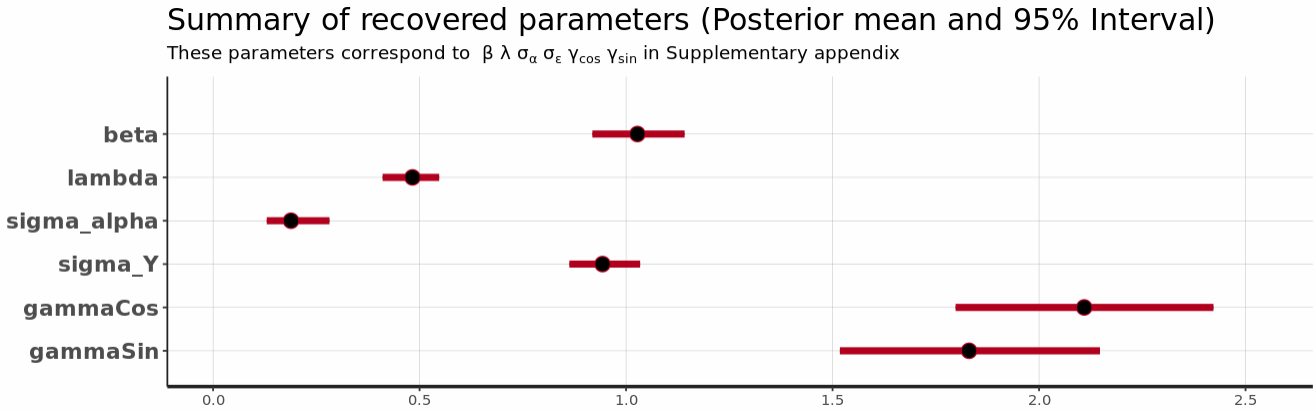


#Posterior distribution of the parameters in transfer function (1,0)

post <- rstan::extract(fit3, pars = c("beta", "lambda"), permuted = TRUE, inc_warmup = FALSE)

betaPosterior = post[[1]]

lambdaPosterior = post[[2]]

# Plot Impulse response function (IRF)

h <- 8 # number of lags to display, increase for long lag

# IRF, mean, lower and uppfer CI

irfWeight <- data.frame(weekLag = 1:h, lo = rep(NA, h), mean = rep(NA, h), hi = rep(NA, h))

# Time t

irfWeight[1, ] <- c(1, quantile(betaPosterior , c(0.025)), mean(betaPosterior), quantile(betaPosterior , c(0.975)))

# time t+(2:h)

for(i in 2:h){irfWeight[i, ] <- c(i, quantile(beta * {lambdaPosterior^(i-1)}, c(0.025, 0.5,0.975)))}

# Plot IRF

irfWeight$lagTrue <- lagFunction[1:h]

scaleFUN <- function(x) sprintf("%.1f", x)

p <- ggplot(data = irfWeight, aes(x=(weekLag), y=mean, linetype = "est")) +

geom_line() +

theme_classic() +

xlab("Lag") + ylab("Change of outcome") +

geom_ribbon(aes(ymin=lo, ymax=hi), linetype=2, alpha=0.1) +

scale_y_continuous(labels=scaleFUN) +

theme(axis.text = element_text(color = "black")) +

ggtitle("Impulse reponse function (Black) \n with pointwise 95% credible range (grey band). \n compared to true lag function (red dotted line)")

# Add the true lag function

p + geom_line(data = irfWeight, aes(x = weekLag, y = `lagTrue`, linetype="true"), ) +

scale_linetype_manual(name = 'Legend', values=c("est" = "solid", "true" = "dashed"), labels = c('Estimated','True'))


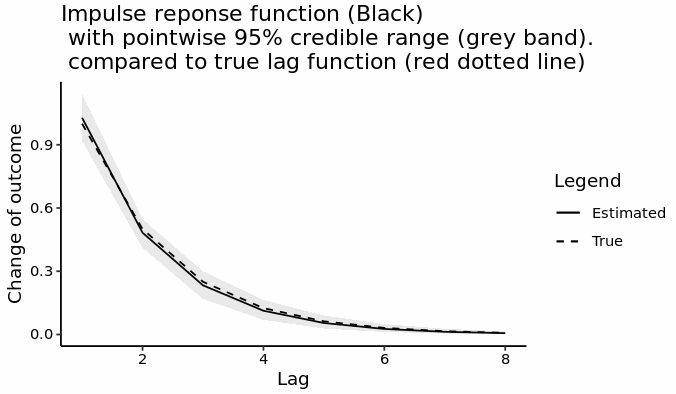


### Posterior fit check, comparison of fitted and true value

post <- rstan::extract(fit3, pars = "yHat")

mean <- apply(post$yHat, 2, mean)

lo <- apply(post$yHat, 2, quantile, 0.025)

hi <- apply(post$yHat, 2, quantile, 0.975)

plot(lo, type = "l", lty = "dotted", ylim = range(c(mean, lo-1, hi +1)), ylab = expression(Y[t]), xlab = "Time")

lines(hi, lty = "dotted")

lines(mean, type = "l", lwd = 0.5)

points(y, pch = 3, cex = 0.5, col = "blue")

title(main = "Fitted mean of outcome (solid line) \n 95% credible interval (dotted line) \n Observed outcome (cross)")

### Posterior fit check, comparison of fitted and unobserved (true) value of intercept

dev.off()

post <- rstan::extract(fit3, pars = "alpha", permuted = TRUE, inc_warmup = FALSE)

mean <- apply(post$alpha, 2, mean)

lo <- apply(post$alpha, 2, quantile, 0.025)

hi <- apply(post$alpha, 2, quantile, 0.975)

plot(lo, type = "l", lty = "dotted", ylim = range(c(mean, lo, hi)), ylab = expression(alpha[t]), xlab = "Time")

lines(hi, lty = "dotted")

lines(mean, type = "l", lwd = 1)

points(alpha, pch = 3, cex = 0.8, col = "blue")

title(main = "Posterior mean of intercept (solid line) \n 95% credible interval (dotted line) \n True intercept (cross)")


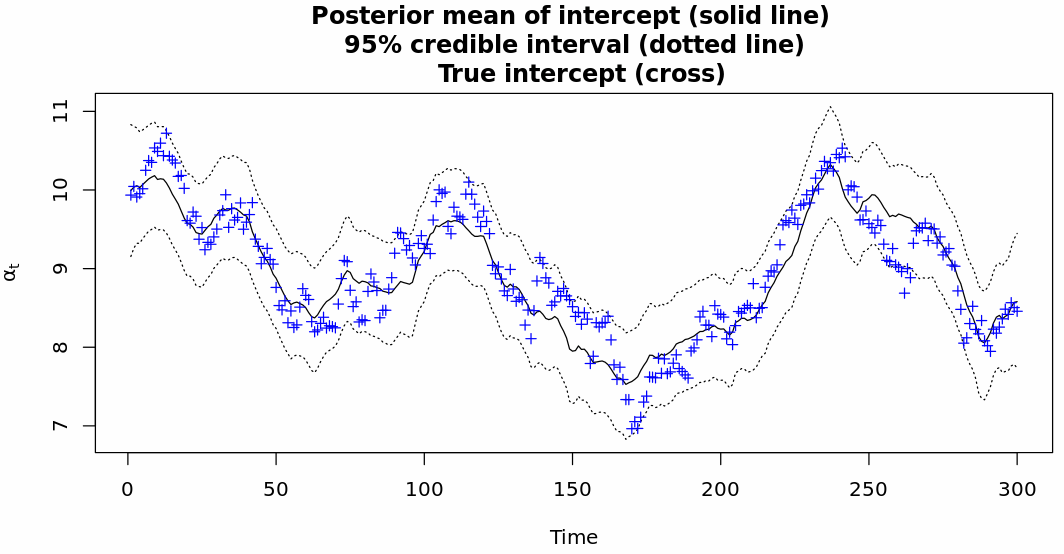


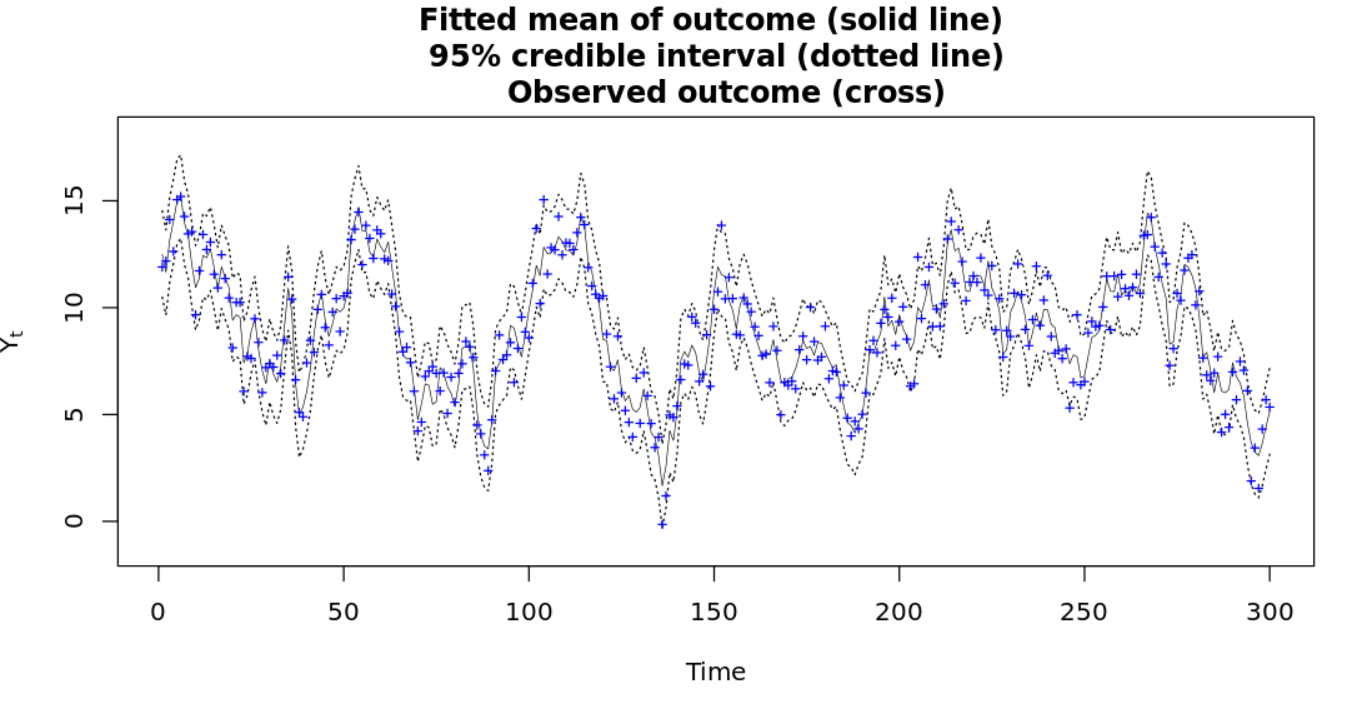


--- End of demo R File ----

// Stan code for 1st order transfer function to capture Koyck lag, with intercept and season wave

// <- double slash indicates comment in .stan script file

// filename: testModel_alphaLevel_season_koyck.stan

data {

int<lower=0> T; // Number of time periods

real y[T]; // Outcome vector

real x[T]; // Exposure vector

real weekIndex[T]; // Week index vector need to create season wave

}

transformed data{

// This section defines season wave

vector[T] week_times_pi_freq;

for (i in 1:T) week_times_pi_freq[i] = 2*weekIndex[i]*pi()/52.4;

}

parameters {

real <lower=0> sigma_Y; // Standard Deviation (SD) for observation error

vector[T] alpha;

real <lower=0> sigma_alpha; // SD of randomly evolving intercept- stochastic level

real beta; // Immediate effect of explanatory variable

real <lower = 0, upper = 1> lambda; // Koyck lag coefficient. In this study, it is constrained to be 0 < lamgda < 1 to allow the association decays to zero

// Season effects

real gammaSin;

real gammaCos;

}

transformed parameters { # Any statements that do not assign random varaibles can be placed here

vector[T] wave; // Seaseon wave

vector[T] E; // Structural varaible of transfer function

// Initialization

E[1] = beta*x[1];

for (t in 2:T){ // Evolution of transfer function (note that error term was not added to this evolution, but it can be added)

E[t] = lambda * E[t-1] + beta*x[t];

}

// Linear combination of season wave - in this study, time-fixed coefficient gammas

for (i in 1:T){

wave[i] = gammaSin*sin(week_times_pi_freq[i]) + gammaCos*cos(week_times_pi_freq[i]);

}

}

model { //Priors, outcome model, and evolution of level

//priors and initial values, can also explore smaller and larger values of scale as sensivity analysis - see manuscript

sigma_Y ~ cauchy(0, 5);

alpha[1] ~ normal(0, 5);

sigma_alpha ~ normal(0, 1);

beta ~ normal(0, 5);

lambda ~ uniform(0, 1);

gammaSin ~ normal(0, 5);

gammaCos ~ normal(0, 5);

// Evoluation of the level intecept

//for(t in 2:T)alpha[t] ~ normal(alpha[t-1], sigma_alpha); // bit slower approach in Stan, not vetocized

alpha[2:T] ~ normal(alpha[1:(T - 1)], sigma_alpha);//this is faster

// The outcome from intercept, lag structure, season and noise

for(t in 1:T) y[t] ~ normal(alpha[t] + E[t] + wave[t], sigma_Y);

}

// generate predictive distribution and log likelihood

generated quantities {

real yHat[T];

real log_lik [T];

for (t in 1:T){

yHat[t] = normal_rng(alpha[t] + E[t] + wave[t] , sigma_Y);

log_lik[t] = normal_lpdf(y[t] | alpha[t] + E[t] + wave[t], sigma_Y);

}

}

// END OF Stan code ------------------------------------------------------------------------------------------------------------------------------


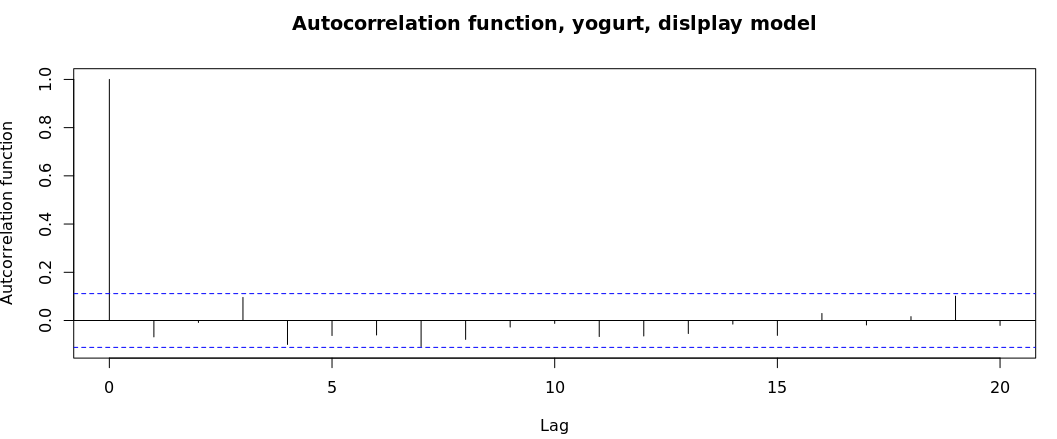


Supplementary eFigure 4. Autocorrelation function of residuals, indicating that there is no significant correlation in time-series of residuals (Montreal, Canada, 2008-2013).

# References for Supplementary Materials

1. Ravines RR, Schmidt AM, Migon HS. Revisiting distributed lag models through a Bayesian perspective. Appl Stoch Models Bus Ind. 2006;22(2):193–210.

2. Box GEP, Jenkins GM, Reinsel GC, Ljung GM. Chapter 11: Transfer function models. In: Time Series Analysis: Forecasting and Control. 5th ed. Hoboken, New Jersey: Wiley; 2015.

3. West M, Harrison J. Bayesian forecasting and dynamic models. Second edition. New York: Springer; 1997. (Springer series in statistics).

4. Helfenstein U. The use of transfer function models, intervention analysis and related time series methods in epidemiology. Int J Epidemiol . 1991;20(3):808–15.

5. Schaffer AL, Dobbins TA, Pearson SA. Interrupted time series analysis using autoregressive integrated moving average (ARIMA) models: a guide for evaluating large-scale health interventions. MC Med Res Methodol. 2021 Mar 22;21(1):58.

6. Peng RD, Dominici F, Welty LJ. A Bayesian hierarchical distributed lag model for estimating the time course of risk of hospitalization associated with particulate matter air pollution. Journal of the Royal Statistical Society: Series C (Applied Statistics). 2009;58(1):3–24.

7. Welty LJ, Peng RD, Zeger SL, Dominici F. Bayesian distributed lag models: estimating effects of particulate matter air pollution on daily mortality. Biometrics. 2009 Mar;65(1):282–91.

8. Hendel I, Nevo A. The post-promotion dip puzzle: what do the data have to say? Quantitative Marketing and Economics. 2003 Dec 1;1(4):409–24.

9. Saha MV, Davis RE, Hondula DM. Mortality displacement as a function of heat event strength in 7 us cities. American Journal of Epidemiology. 2014 Feb 15;179(4):467–74.

10. Lee A, Mhurchu CN, Sacks G, Swinburn B, Snowdon W, Vandevijvere S, et al. Monitoring the price and affordability of foods and diets globally. Obes Rev. 2013 Oct 1;14:82–95.

11. Chollet M, Gille D, Schmid A, Walther B, Piccinali P. Acceptance of sugar reduction in flavored yogurt. Journal of Dairy Science. 2013 Sep 1;96(9):5501–11.

12. Nielsen. Retail Measurement: in-House Retail Experts [Internet]. [cited 2018 Apr 12]. Available from: http://www.nielsen.com/id/en/solutions/measurement/retail-measurements

13. Hecht AA, Perez CL, Polascek M, Thorndike AN, Franckle RL, Moran AJ. Influence of food and beverage companies on retailer marketing strategies and consumer behavior. Int J Environ Res. 2020 Jan;17(20):7381.

14. Petris G, Petrone S, Campagnoli P. Dynamic linear models with R. New York: Springer-Verlag; 2009. (Use R!).

15. Vehtari A, Gelman A, Gabry J. Practical Bayesian model evaluation using leave-one-out cross-validation and WAIC. Stat Comput. 2017 Sep 1;27(5):1413–32.

16. Stan Development Team. RStan: the R interface to Stan [Internet]. 2020 [cited 2020 Nov 20]. Available from: http://mc-stan.org/

17. Gelman A. Prior distributions for variance parameters in hierarchical models. Bayesian Analysis. 2006;1(3):515–33.
